# Anatomic Demarcation by Positional Variation in Fibroblast Gene Expression Programs

**DOI:** 10.1371/journal.pgen.0020119

**Published:** 2006-07-28

**Authors:** John L Rinn, Chanda Bondre, Hayes B Gladstone, Patrick O Brown, Howard Y Chang

**Affiliations:** 1 Program in Epithelial Biology, Department of Dermatology, Stanford University School of Medicine, Stanford, California, United States of America; 2 Department of Biochemistry, Stanford University School of Medicine, Stanford, California, United States of America; 3 Howard Hughes Medical Institute, Stanford University School of Medicine, Stanford, California, United States of America; MRC Human Genetics Unit, United Kingdom

## Abstract

Fibroblasts are ubiquitous mesenchymal cells with many vital functions during development, tissue repair, and disease. Fibroblasts from different anatomic sites have distinct and characteristic gene expression patterns, but the principles that govern their molecular specialization are poorly understood. Spatial organization of cellular differentiation may be achieved by unique specification of each cell type; alternatively, organization may arise by cells interpreting their position along a coordinate system. Here we test these models by analyzing the genome-wide gene expression profiles of primary fibroblast populations from 43 unique anatomical sites spanning the human body. Large-scale differences in the gene expression programs were related to three anatomic divisions: anterior-posterior (rostral-caudal), proximal-distal, and dermal versus nondermal. A set of 337 genes that varied according to these positional divisions was able to group all 47 samples by their anatomic sites of origin. Genes involved in pattern formation, cell-cell signaling, and matrix remodeling were enriched among this minimal set of positional identifier genes. Many important features of the embryonic pattern of *HOX* gene expression were retained in fibroblasts and were confirmed both in vitro and in vivo. Together, these findings suggest that site-specific variations in fibroblast gene expression programs are not idiosyncratic but rather are systematically related to their positional identities relative to major anatomic axes.

## Synopsis

A major question in developmental biology is, How do cells know where they are in the body? For example, skin cells on the scalp know to produce hair, and the skin cells on the palms of the hand know not to make hair. Overall, there are thousands of different cell types and each has a unique job that is important to overall organ function. It is critical that, as we grow and develop, each of these different cells passes on the proper function from generation to generation to maintain organ function. In this study, the authors present a model that explains how cells know where they are in the body. By comparing cells from 43 unique positions that finely map the entire human body, the authors discovered that cells utilize a ZIP-code system to identify the cell's position in the human body. The ZIP code for Stanford is 94305, and each digit hones in on the location of a place in the United States; similarly, cells know their location by using a code of genes. For example, a cell on the hand expresses a set of genes that locate the cell on the top half of the body (anterior) and another set of genes that locates the cell as being far away from the body or distal and a third set of genes that identifies the cell on the outside of the body (not internal). Thus, each set of genes narrows in on the cell's location, just like a ZIP code. These findings have important implications for the etiology of many diseases, wound healing, and tissue engineering.

## Introduction

The problem of how genetic information gives rise to the spatial organization has long intrigued developmental biologists [1–3]. While cellular differentiation addresses the control of expression of specific genes within a cell, pattern formation addresses the spatial arrangement of distinct cell types. A major mechanism of pattern formation in the embryo is the use of positional information. By linking differentiation programs to cell positions on a coordinate system, an assembly of cells can be programmed to develop into well-defined spatial patterns that are not easily perturbed by the removal or addition of cells. While spatial boundaries are first defined during embryonic development, these spatial patterns of cellular specialization also need to be maintained throughout adulthood as the tissues undergo continual self-renewal. In contrast to embryonic development, the higher-order patterns of cellular specialization in adult animals and mechanisms of their maintenance are less well understood.

The skin is a prime example of an organ with clear spatial patterns of morphologic and functional specialization. For instance, terminal hairs grow on top of the head but not on the palm of the hand. Moreover, a large number of skin diseases show striking specificity for particular anatomic site [4]. In all epithelial organs, differentiated epithelial cells are juxtaposed to stromal tissue, which consists of fibroblasts and the extracellular matrix traversed by blood and lymphatics vessels, nerves, and intermingled with leukocytes. During development, reciprocal epithelial-mesenchymal interactions pattern a wide variety of epithelial tissues as diverse as skin, lung, and intestine (reviewed in [5]). Classic embryological studies demonstrated that a primary mesenchymal or dermal signal is often responsible for conveying positional identity during embryogenesis. Transplantation of wing epithelium to leg mesenchyme transforms the development of feathers, a type of epithelial derivative, to the development of scales [6]. Thus, one or more stromal cell types must encode position information in order to convey proper inductive signals to epithelial cells.

Fibroblasts are an important constituent of stroma and play key roles in development, repair, and disease. Fibroblasts synthesize extracellular matrix components throughout the body, and orthotopic fibroblasts can substitute for mesenchyme in site-specific epithelial induction in several settings [7–9]. For instance, dermal papilla cells from skin-bearing terminal hairs (e.g., on the scalp) signal to the follicular epithelial cells to regulate the hair cycle, whereas the dermis of glabrous skin (e.g., the palm of the hand) activates alternative epithelial fates [10]. Moreover, fibroblasts play important roles during wound healing and are involved in the pathogenesis of many diseases that result in scarring and fibrosis [11,12]. Therefore, the functional diversity of fibroblasts is likely to play important roles in specifying the developmental and physiologic specialization of many tissues.

Fibroblasts are traditionally defined by their spindle-shaped morphology, ability to adhere to plastic substratum in culture, and absence of additional lineage-specific markers. Although fibroblasts from different anatomic sites are morphologically similar, we previously showed that fibroblasts from several sites exhibit large-scale differences in their gene expression programs depending on their anatomic site of origin [13]. Fibroblasts from different anatomic sites may even be considered distinct cell types because their gene expression programs are as diverse as cells from different hematopoietic lineages. Notably, adult fibroblasts retained features of the embryonic HOX code, the spatial pattern of expression of a family of transcription factors that delineate positional identity [13]. These findings raise the possibility that fibroblasts, in adult tissues, might have an important role in encoding positional identity.

As Lewis Wolpert noted in his classic treatise on pattern formation, the apparent organization of specialized cell types in a system can be achieved by several different mechanisms [2]. On one hand, cell types can be uniquely specified by local interactions (such as by lineage or sequential inductive events) and subsequently placed based on mutual attraction or repulsion. Alternatively, pattern can be achieved by cells interpreting their positions relative to reference points and adopting specific differentiation programs based on their positional identity within a coordinate system. A particularly attractive and distinctive feature of the positional identity model is the parsimonious use of molecular entities to construct the system, leading to universality of the coordinate system. For example, the same reference points can be used to specify the proximal-distal axis of the upper and lower limbs even though the limbs are spatially distant from each other. Similarly, the same reference points can pattern the anterior-posterior axis of the trunk as well as distinguish the upper and the lower limbs.

We hypothesized that these models of pattern formation can be distinguished by comparing the global gene expression profiles of cells distributed throughout the body. Pattern formation by local interactions predicts that the similarity of gene expression profiles of cells will be inversely related to the distance of their anatomic sites of origin from each other. Cells more closely spaced from each other are more likely to have shared local interactions, and therefore may have greater similarity to each other in gene expression. A second consequence of cell specification by unique local interactions is that spatially distant cells are expected to exhibit complex differences in gene expression: Each pairwise comparison may reveal a different set of genes that are differentially expressed. In contrast, the positional identity model predicts that gene expression profiles of cells will be related to their positions relative to anatomic axes, but less so by spatial distance. For instance, cells from the hand and feet may share a distinct gene expression signature that reflects their distal position along the limbs even though hand and feet are spatially far apart.

In this study, we present and analyze the global gene expression patterns of 47 fibroblast populations from 43 anatomic sites that span the human body. This dense mapping of anatomic sites enabled an unsupervised, agnostic approach to investigate the spatial patterns of fibroblast gene expression within each anatomical structure. Based on the spatial patterns suggested by the unsupervised analysis, we developed a global model and identified specific genes that were predictive of the anatomic site of origin of fibroblasts. We validated this model by comparison to randomized data, cross-validation in independent samples, and in vivo localization analyses. Using this strategy, we found a surprisingly simple organization of fibroblast diversity. Much of the variation in fibroblast gene expression across body sites was accounted for by the site of origin relative to three anatomic divisions: anterior-posterior, proximal-distal, and dermal-nondermal. These results suggest that adult fibroblasts encode positional identity relative to embryonic developmental axes.

## Results

**Figure 1:**
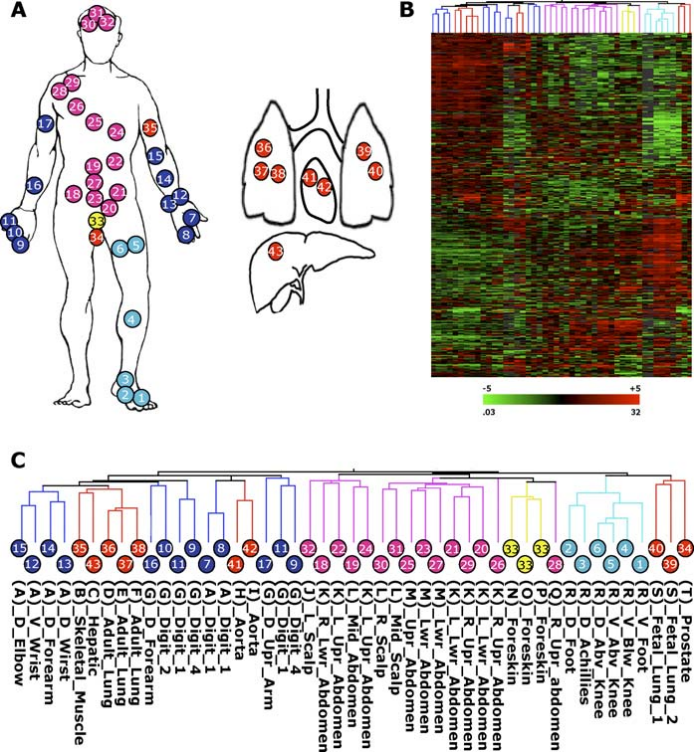
Diversity of Gene Expression in Human Fibroblasts (A) Forty-seven primary fibroblast populations, from 43 unique anatomic sites, were obtained from arm (blue circles), leg (turquoise circles), trunk (pink circles), foreskin (yellow circles), and internal organs (red circles). The cells were derived from 20 different human autopsy or surgery donors identified by the letters A through T in parenthesis. (B) Diversity of gene expression programs in 47 fibroblast populations. Each row represents a gene; each column represents a fibroblast population. The expression level of each gene is represented relative to the median value in all samples; expression levels above and below the global median are denoted by shades of red or green, respectively. The color scale encompasses a range from 32- to 0.03-fold relative to global median transcript level for each gene (+5 to −5 logs on log base 2 scale). Black represents the median expression value; gray represents missing data. (C) Similarity in the global gene expression profiles of 47 fibroblast samples. We used unsupervised hierarchical clustering of 7,580 genes that were reliably measured in 70% of the samples and varied by 3-fold above the global median in at least five (approximately 10%) of the samples. Thirty-five of the 47 samples were placed in clusters composed predominantly of cells from the same anatomic origins (arm, leg, trunk, foreskin, or nondermal).

To characterize the diversity of human fibroblasts, we cultured 47 primary fibroblast populations from 43 anatomical sites (Figure 1A and Dataset S1). These sites spanned several anatomical structures of the human body, including arm, hand, leg, foot, chest, and internal organs. We collected fibroblasts from multiple sites of the same donor, and from the same sites of multiple donors. We only used fibroblast cultures that exclusively expressed vimentin (a mesenchymal marker) and did not express lineage-specific markers for epithelial cells (cytokeratin), smooth muscle (desmin), endothelial cells (PECAM), neural cells (GFAP), or macrophages (CD11b). To explicitly test the capacity of cells to maintain site-specific differentiation, each primary culture was propagated under identical conditions in vitro for at least five passages to remove the effect of their native environments.

The global gene expression profile of each fibroblast population was assayed by hybridization of labeled messenger RNA to cDNA microarrays containing 41,121 elements, representing 24,421 unique genes. Over 1 million gene expression measurements were made. We focused on genes that were reliably measured in at least 70% of the samples and whose expression varied at least 3-fold from the mean across all samples in five or more samples; 7,580 genes passed these criteria. To explore the relationships among the 47 samples, we used unsupervised hierarchical clustering to group the samples and genes by similarity. Fibroblasts were generally grouped with other fibroblasts from the same anatomic sites of origin (Figure 1B and 1C). Thirty-five of 47 samples were placed within clusters that were composed predominantly of other fibroblasts from the same anatomic origin. For instance, six of six fibroblasts from the lower limb were grouped in one cluster; similarly, chest (14 of 15), foreskin (three of three), arm (seven of 13), and nondermal (five of ten) fibroblasts were grouped in clusters that had a majority of fibroblasts from the same anatomic site. This pattern of anatomic-specific grouping was apparent among fibroblasts derived from different donors. In instances where we had fibroblasts from multiple sites from the same donor, fibroblasts were grouped with cells from the same anatomic site from other individuals rather than with other cells from the same donor. Thus, the effect of donor-to-donor variation on global gene expression patterns of cultured fibroblasts was small compared to the effect of variation in anatomic origin. These results confirm, on a much larger scale, our previous observation that fibroblasts in different anatomic sites are molecularly distinct differentiated cells [13].

### Fibroblasts from Several Anatomic Structures Exhibit Compartmental Patterns of Gene Expression

We looked for organizing principles that might underlie the significant diversity of gene expression patterns among fibroblasts. Because the scope of diversity might be relatively limited within a distinct anatomic structure (such as a limb), we reasoned that systematic analysis of differences in gene expression within a structure might reveal simple rules underlying the patterns of expression variation. Therefore, we first examined the diversity of fibroblast gene expression within each anatomical structure using unsupervised hierarchical clustering, and we then determined whether the same principles of organization were applicable to the whole body.

**Figure 2:**
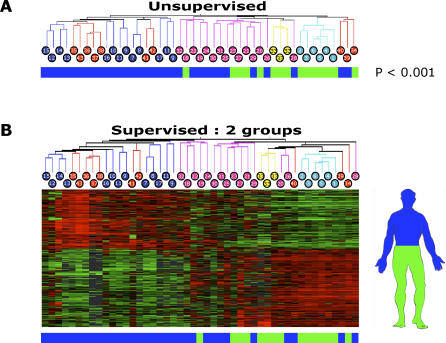
A Gene Expression Signature Divides Fibroblasts from Anterior and Posterior Sites of the Human Body (A) Unsupervised hierarchical clustering separated fibroblasts from above and below the umbilicus. Blue bars indicate anterior (rostral) samples; green bars indicate posterior (caudal) samples. (B) Supervised analysis with SAM using all samples identifies two distinct gene expression profiles corresponding to the origin of most samples being anterior and posterior to the umbilicus (FDR = 1%).

First, we noticed that there was a salient separation of fibroblasts from the top half (anterior or rostral) versus the bottom half (posterior or caudal) of the human body based on their unsupervised gene expression profiles (Figure 2A and Dataset S2). In addition to the clustering based on anatomic sites noted above, the first bifurcation of the dendrogram identified two groups of fibroblasts that were strongly correlated with their site of origin relative to the anterior-posterior axis. Twenty of 20 fibroblasts from sites above the umbilicus were in the left cluster, and 15 of 27 fibroblasts from below the umbilicus were in the right cluster (*p* < 0.001, χ^2^ test). Supervised analysis using the permutation-based algorithm Significance Analysis of Microarrays (SAM; http://www-stat.stanford.edu/~tibs/SAM) revealed 1,172 genes demonstrating an anterior-posterior expression pattern separated at the umbilicus (false discovery rate [FDR] = 1%, Figure 2B). Intriguingly, several samples from near the umbilicus and shoulder demonstrated intermediate expression patterns and were misclassified using this gene expression signature as the criterion. As a sample was located further from the umbilicus, the more it adhered to the anterior-posterior expression pattern.

**Figure 3:**
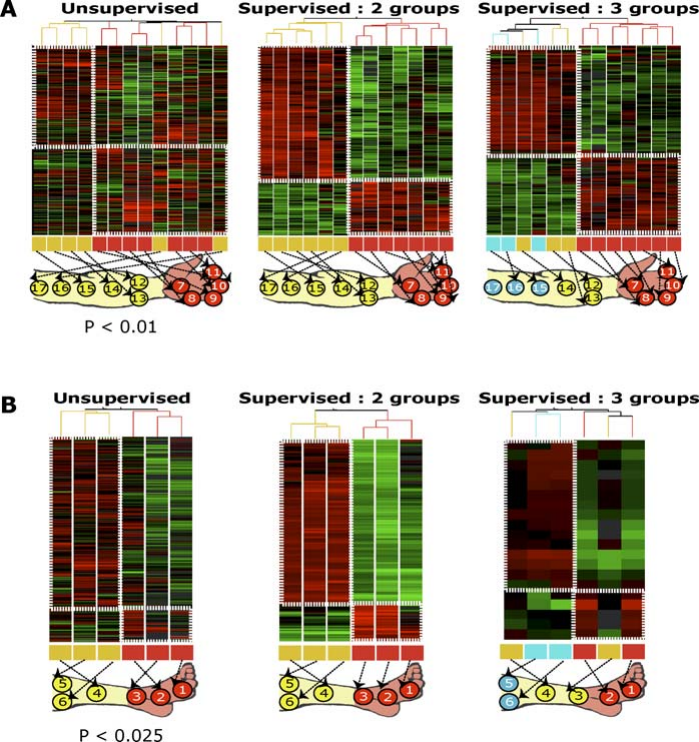
Fibroblasts from Limbs Exhibit Binary Gene Expression Signatures that Demarcate Proximal-Distal Position (A) Unsupervised hierarchical clustering of all upper limb-derived fibroblasts (left) demonstrates differential gene expression between the proximal and distal upper limb fibroblasts. Supervised analysis with SAM (FDR = 5%) revealed two strong gene expression patterns that separate all proximal and distal samples (middle). A three-class analysis (right) did not reveal a third gene expression pattern. (B) Unsupervised hierarchical clustering (left) of lower limb-derived fibroblasts also exhibited a division of proximal and distal lower limb fibroblasts. Supervised analysis with SAM (FDR = 5%) revealed a binary expression pattern separating the proximal and distal samples (middle). Organizing the leg into three segments and using a corresponding multiclass analysis failed to reveal a third gene expression pattern (right).

Second, unsupervised analysis of gene expression profiles of fibroblasts from the upper and lower limbs revealed biphasic patterns that distinguished cells along the proximal-distal axis (Figure 3 and Dataset S3). In the first bifurcation of the dendrogram comparing gene expression of fibroblasts from the upper limb, seven of seven fibroblasts from sites distal to the wrist (e.g., hand and fingers) were in the right cluster, and four of six fibroblasts proximal to the wrist were in the left cluster (*p* < 0.01, χ^2^ test). Similarly, the first bifurcation of the comparison of fibroblasts from the lower limb separated three of three fibroblasts distal to the ankle in the right cluster from three of three fibroblasts proximal to the ankle in the left cluster (*p* < 0.025, χ^2^ test). The extent of grouping by position along the proximal-distal axis in both limbs is significantly greater than expected by chance alone (*p* < 0.001). Importantly, fibroblasts from hair-bearing skin from distal limbs (e.g., the back of the hand, finger, and feet) exhibited expression signatures remarkably similar to those of fibroblasts from cognate distal non–hair-bearing (glabrous) skin (e.g., palm, finger, and sole) but distinct from those of fibroblasts derived from proximal limbs. These results indicate that the distal pattern is not simply a reflection of origin from hair-bearing versus glabrous skin but is more consistently related to position within the limb.

Using SAM, we identified a set of genes that were significantly differentially expressed between the proximal and distal samples in the arm and leg, respectively. In both cases we identified genes with a sharply bounded expression pattern segmenting the proximal and distal samples (Figure 3, middle panels). Because this first proximal-distal boundary corresponded to the embryonic domain of autopod (hand and foot) from the remainder of the limb, we asked if additional gene expression signatures distinguished fibroblasts derived from embryonic domains of stylopod (upper arm and thigh) versus zeugopod (lower arm and calf). Surprisingly, in both the arm and leg, explicit search for this additional expression signature did not provide evidence beyond the two predominant patterns of gene expression (Figure 3, right panels). We also did not find genes that were expressed in fibroblasts according to a gradient along the limbs.

Third, we found characteristic differences between the expression profiles of fibroblasts cultured from dermal and nondermal sites, such as the lung, heart, liver, and prostate. Figure S1 shows 396 genes (FDR = 0.1%) that demonstrate significant differential expression between fibroblasts from dermal versus nondermal sites.

Surprisingly, we were unable to uncover strong evidence for genes with differential expression related to fibroblast position along the dorsal-ventral axis. Comparison of two independent sets of donor-matched fibroblasts derived from the dorsal and ventral aspects of limbs identified one gene, encoding *Protocadherin 18,* that were more highly expressed in cells from the dorsal sites. This gene was considered significant in each comparison (FDR > 1%) and the same gene showed the greatest differential expression in both comparisons. Moreover, having the same gene in both comparisons is not expected by chance (*p* < 0.0002, hypergeometric distribution). Genes with well-known differential expression along the dorsal-ventral axis during embryogenesis (*TWIST1, RHBDL2, RHBDF1, ENGRAILED, SNAIL,* and *BMP4*) also did not exhibit differential expression along the dorsal-ventral axis in our fibroblast samples. These results suggest that there are few if any robust gene expression differences between fibroblasts cultured from dorsal and ventral sites, which is consistent with prior studies demonstrating epithelial dominance in dictating dorsal-ventral fates in the limb [14,15].

In summary, by organizing fibroblast samples based on their global gene expression profiles, we observed grouping of fibroblasts based on their site of origin relative to three anatomic divisions: anterior-posterior, proximal-distal, and dermal nondermal.

### Recurrence of Gene Expression Signatures Reflecting Positional Identity throughout the Body

**Figure 4:**
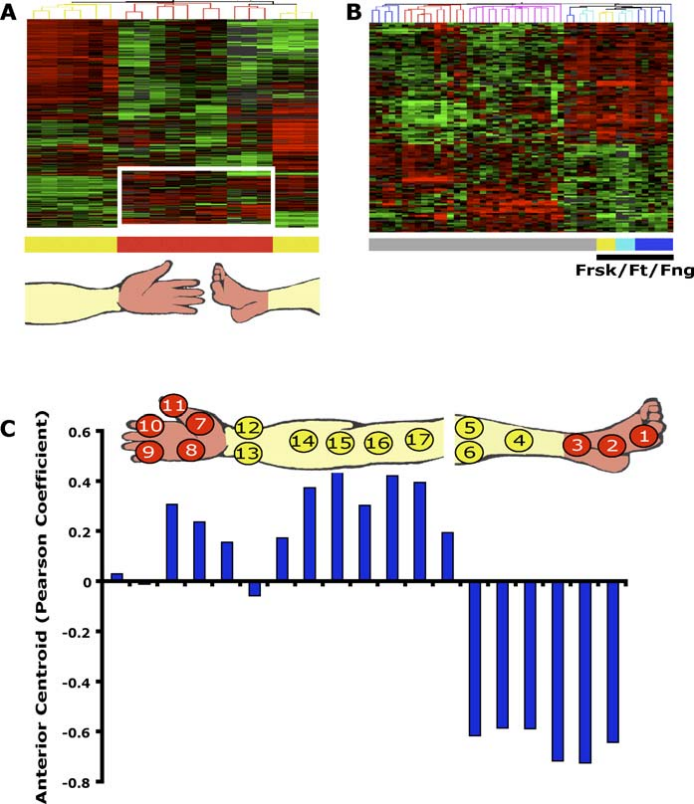
Gene Expression Signatures Related to Anatomic Divisions Recur throughout the Human Body (A) Hand and foot fibroblasts share a distal-specific gene expression signature. Hierarchical clustering of arm and leg samples together, using genes that are differentially expressed between the proximal and distal regions of the arm and leg; 98 genes that are relatively induced in distal-derived fibroblasts are highlighted by the white box. (B) Hierarchical clustering of all 47 samples using the 98 distal genes revealed that foreskin (frsk) samples were most closely related to the feet (ft) and finger (fng) samples, indicating that the “distal” gene expression pattern of these genes is repeated in the feet, fingers, and foreskin. (C) Distinction of arm and leg fibroblasts by an anterior gene centroid. We created an averaged gene expression pattern, termed a centroid (methods), from the gene expression profiles of anterior samples from head, chest, and trunk. The anterior centroid is then compared to the expression profile of each arm and leg sample for similarity (using noncentered Pearson correlation). The anterior centroid was positively correlated with all except two profiles of arm fibroblasts and negatively correlated with all leg fibroblasts. We also calculated a posterior centroid from trunk samples below the umbilicus, and it negatively correlated with all arm samples and positively correlated with all leg samples (data not shown).

Because fibroblasts demonstrated differential gene expression along the proximal-distal axis in separate comparisons of the upper and lower limbs, we next asked if the distinction of proximal-distal position in upper and lower limbs involved the same genes. We used hierarchical clustering to organize all limb-derived fibroblasts based on the expression profile of all genes that were differentially expressed in a proximal-distal pattern in either upper or lower limb. Fibroblasts from distal sites in the upper and lower limbs (i.e., the hand and feet) had substantial similarity in their gene expression pattern that distinguished them from fibroblasts of proximal limb origin (Figure 4A and Dataset S4). Since 98 genes shared a similar “distal” expression pattern in the fibroblasts derived from hand and foot (Figure 4A, box), we investigated whether other fibroblast samples also shared this distal expression profile. Using these 98 distal genes to hierarchically cluster all 47 fibroblast samples revealed a notable similarity of hand, foot, and foreskin fibroblasts in their expression of these distal genes, and these three types of fibroblasts were consequently grouped together (Figure 4B). These observations suggest that the spatial pattern of fibroblast differentiation in the upper and lower limbs is in part defined by position within each structure rather than simply being defined by their anatomic structure of origin. Moreover, a presumptive distal gene expression signature identified in limb fibroblasts is shared between these distinct sites and may also characterize other developmentally distal elements such as the foreskin.

An anterior-posterior expression pattern also appears to be a recurring theme in the gene expression programs of fibroblasts. We identified a set of genes that were differentially expressed along the anterior-posterior division using only fibroblasts from the trunk and asked whether the same set of genes could distinguish fibroblasts from upper versus the lower limb. Almost all fibroblasts derived from the upper limb showed greater similarity to the anterior gene expression signature while all fibroblasts derived from lower limb had greater similarity to the posterior gene expression signature (Figure 4C and Datasets S1–S7). We also performed a cross-validation test where we attempted to predict the site of origin of held-out fibroblast samples based on gene expression signatures learned from the remaining fibroblasts. Overall, we correctly predicted the positional origin (anterior or posterior, proximal or distal) of the test fibroblast samples with 80% accuracy (*p* = 0.0046, binomial test; Figure S2). Thus, there is a remarkable generality of the gene expression signatures that characterize positional identity of fibroblasts within specific anatomic structures.

### A Model of Fibroblast Differentiation based on Anatomic Divisions of Gene Expression Patterns

**Figure 5:**
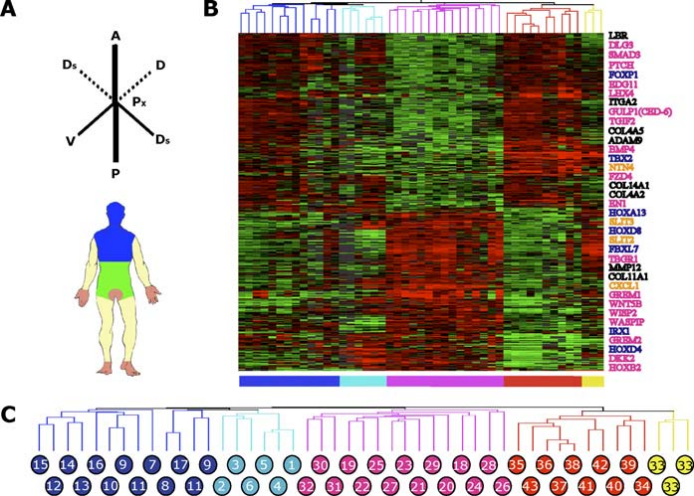
Genes that Vary According to Three Binary Anatomical Divisions Organize Fibroblasts by Their Anatomic Sites of Origin throughout the Body (A) A positional model where each fibroblast sample belonged to one segment. A multiclass analysis of the 47 samples, classified according to an Anterior (blue), Posterior (green), Proximal (yellow), Distal (red), and nondermal (not shown). (B) Heat map of the 337 genes that overlapped between the model in (A) and in one or more of the comparisons within local anatomic structures in Figures 2 to 4. These 337 genes contained many transcription factors (gene names in blue), components and enzymatic modifiers of the extracellular matrix (gene names black), signaling proteins in development (gene names in pink), and guidance molecules in cell migration (gene names in orange). (C) The selected 337 genes grouped 47 of 47 of the fibroblast samples within clusters composed exclusively of samples from the same anatomical regions.

Our local and global analyses revealed that the spatial patterns of fibroblast differentiation are associated with gene expression signatures related to anterior-posterior, proximal-distal, and dermal-nondermal divisions. These observations raise the possibility that fibroblast differentiation is governed in part by the combinatorial superposition of gene expression programs representing broad anatomic divisions, to confer distinct identities to the cells in each unique anatomic structure. To test this hypothesis, we mapped onto the body the positional boundaries suggested by the three distinct binary divisions of gene expression signatures (Figure 5A and Dataset S5). Each fibroblast sample was assigned to one of five classes (proximal, distal, anterior, posterior, or nondermal); we then performed a multiclass comparison to identify genes whose variation in expression most strongly distinguished one or more of the classes (see Materials and Methods). We further required the genes to have differential expression in at least one of three anatomic divisions described above. Modeling the samples in this way allowed us to capture gene expression differences that distinguished fibroblast origin locally and were applicable throughout the body.

Three hundred thirty-seven genes met these criteria and appear enriched for several functional themes (Figure 5B). The 337 genes identified by position-specific variation in expression included many transcription factors involved in specifying positional identity *(HOXA5, HOXA11, HOXA13, HOXD8, TBX2, TBX15, EMX2, FOXF1, FOXP1,* and *TRPS1),* signaling pathways involved in cell fate determination *(WNT1, WNT5b, WISP2, DKK2, GPC6, BMP4, GREM2, SMAD3, PTCH,* and *GULP1),* guidance molecules in cell migration *(NTN4, SLIT2, SLIT3, CXCL1, CCRL1,* and *THBS1),* extracellular matrix components *(COL4A2, COL4A5, COL8A2, COL10A1, COL11A1, FN1, EMILIN2,* and *HS3ST3B1),* and enzymes for remodeling the extracellular matrix *(ADAM9, ADAMTS5, MMP3, CSTE, CTSZ, PLAT,* and *PLAU)* (Figure 5B). These biological themes are also supported by a quantitative enrichment analysis based on the public annotation Gene Ontology [16] (Table S1). The different combinations of these transcription factors, signaling proteins, and matrix environments could potentially regulate position-specific differentiation of fibroblasts and also provide signals to influence site-specific fates of neighboring cells, an important characteristic for specifying positional information in the stroma.

In addition, hierarchical clustering of all fibroblast samples based on variation in expression of these 337 genes placed all fibroblasts samples (47 of 47) into clusters composed exclusively of their anatomic neighbors (Figure 5B and 5C). The five anatomic clusters defined by their patter of expression of this set of genes were upper limb, lower limb, head and trunk, foreskin, and nondermal tissues. These anatomical groupings were similar to the groups obtained by unsupervised hierarchical clustering of samples using all 7,580 variably expressed genes (Figure 1C), but substantially more fibroblast samples were correctly grouped with their anatomic neighbors when these selected 337 genes were used. This improved organization by anatomic origin suggest that these 337 genes can capture much of the positional information of fibroblasts relative to three anatomic axes.

### Uniqueness of Gene Selection and General Applicability of a Model of Fibroblast Differentiation Based on Positional Identity

**Figure 6:**
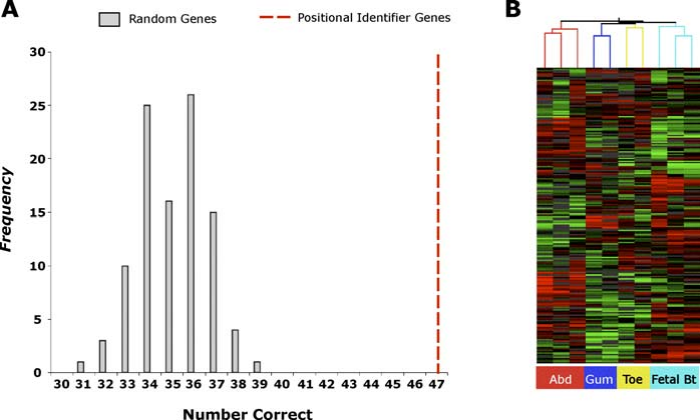
Uniqueness of Gene Selection and General Applicability of a Model of Fibroblast Differentiation Based on Positional Identity along Three Anatomic Divisions (A) Statistical significance of grouping fibroblasts by their anatomic origin using the minimal set of 337 positional identifier genes. One hundred random sets of 337 genes from 7,580 well-measured and variably expressed genes were tested for their ability to organize fibroblasts by anatomic origin using hierarchical clustering; the results are shown as a histogram. We scored the minimum number of branches in the dendrogram that have to be moved to place all the samples into clusters that composed exclusively of samples from each anatomical region. The median number of correctly characterized samples by random gene sets (gray bars) was 35 (12 branch movements), and no random gene set matched the performance of the 337 genes representing positional segments (red dashed line, 47 of 47 correct). The observed result with the 337 genes is seven standard deviations away from the median performance of random gene sets. (B) The 337 genes organized an independent set of fibroblasts by anatomic origin. Using hierarchical clustering, expression patterns of the 337 genes correctly grouped ten of ten new fibroblast samples according to their location in the abdomen (Abd), gum, toe, and fetal buttocks (Fetal Bt).

We performed four analyses to evaluate how well these 337 genes had captured information relevant to specific anatomic structures. First, we compared these 337 genes to 100 different random sets of 337 similarly well-measured and variably expressed genes. Each randomly selected set of 337 genes was used to group the 47 fibroblasts samples by hierarchical clustering, and the accuracy of fibroblast classification relative to their site of origin was scored (see Materials and Methods). We found that it was highly unlikely for a random set of 337 genes to correctly group 47 of 47 fibroblast samples (*p* = 0.00017), and none of the 100 random gene sets approached the accuracy achieved by the 337 genes selected by our model (Figure 6A and Dataset S6). Interestingly, the median number of samples correctly characterized by these random groups of 337 genes was 35, the same number that is observed using all 7,580 well-measured and variably expressed genes. Second, to test the uniqueness of the 337 genes in demarcating positional identity, we removed the 337 genes from the microarray data and repeated each of the local and the global comparisons (see Materials and Methods). The resulting gene set from this repeat selection was substantially less informative in clustering fibroblasts based on their anatomic origins, yielding only 35 of 47 correct (a number that is not different from using all genes or random sets of genes). Together, these analyses suggest that the 337 genes whose variation in expression reflect position along the three anatomic axes are enriched for genes that are uniquely informative of site-specific differentiation of fibroblasts.

Third, we explicitly searched for genes that have exclusive expression among the five “anatomic regions” (upper limb, lower limb, head and trunk, foreskin, and nondermal tissues) and used the expression profile of the resulting genes to organize fibroblast samples. A model of unique specification for each anatomic region would predict that such supervised analysis would efficiently identify the differentially expressed genes that distinguished the five anatomic regions from each other. In contrast, a model of patterning by positional identity predicts that anatomic regions are better described by their position on a coordinate system. Therefore, contiguous anatomic regions are paradoxically better described by identifying the combination of genes that specified the individual axes of the coordinate system. Indeed, the genes that were selected based on expression among the five anatomic regions grouped only 35 of 47 fibroblasts correctly by region, which was the same number achieved by using all genes or randomly selected genes (Figure S3).

Fourth, the anatomic divisions we identified are co-extensive with the body, and therefore even fibroblasts from sites not used in learning these expression profiles may be recognized and grouped with their neighbors. We tested the 337 genes to determine whether their variation in expression can predict anatomical origins of an independent panel of ten fibroblast samples from four anatomic site (abdomen, gum, fetal buttock, and toe). None of the ten fibroblast profiles were used as part of the training, and three of four sites (toe, gum, buttock) were not represented by any samples in the training set. Notably, clustering fibroblasts by expression pattern of the 337 genes correctly grouped all samples from the same anatomic origin next to each other (Figure 6B). These results indicate that variation of gene expression along the three anatomic divisions may be a general property of fibroblasts gene expression programs throughout the body.

### An HOX Code in Adult Fibroblasts

The positional identities of adult fibroblasts raise the question of whether their cognate coordinate system was established during embryonic development. During embryogenesis, expression of specific *HOX* genes demarcates distinct positional identities that lead to site-specific cellular differentiation and tissue morphogenesis. Preservation of the embryonic coordinate system predicts that features of the embryonic HOX code may be systematically maintain in adult fibroblasts.

**Figure 7:**
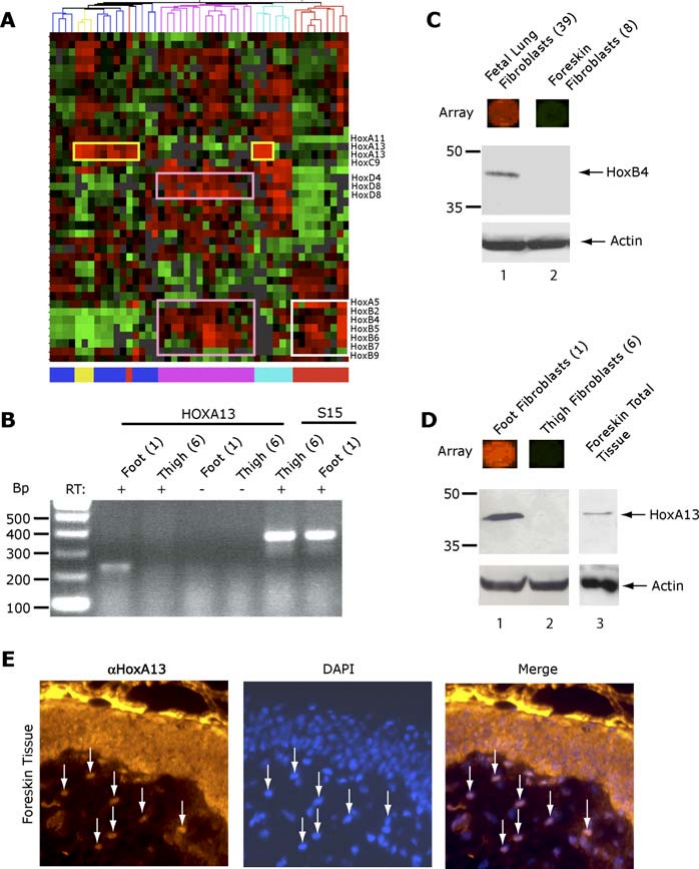
Features of the Embryonic HOX Code Are Maintained in Adult Fibroblasts (A) Expression patterns of 43 homeodomain transcription factors, including 12 HOX genes, organized 43 of 47 the samples by their anatomic origin. *HOXA13* (yellow boxes) is expressed in fibroblasts from distal sites: feet, fingers, foreskin, and prostate. *HOXB2-HOXB9* genes are expressed exclusively in fibroblasts from trunk (pink boxes) and nondermal sites (white box), echoing their expression pattern during early embryogenesis. HOXD genes are expressed in the trunk (and proximal leg) but not in the nondermal samples (top pink box). (B) Validation of *HOXA13* mRNA expression in fibroblasts from distal foot (culture 1), but not fibroblasts from thigh (culture 6). Non-RT negative controls (lanes 3 and 4) and ribosomal protein S15 loading control (lanes 5 and 6) are also shown. (C) Immunoblotting confirmed HoxB4 protein expression in fetal lung fibroblasts but not in foreskin fibroblasts. Raw microarray images shown above indicate RNA expression level. (D) Immunoblotting confirmed HoxA13 protein expression in foot fibroblasts and total foreskin tissue, but not in thigh fibroblasts. Raw microarray images shown above indicate RNA expression level. (E) Immunofluorescence of HoxA13 protein expression in foreskin tissue sections. Nuclei are highlighted by DAPI staining.

We found that several aspects of *HOX* gene expression pattern paralleled the patterns of these genes in early embryogenesis (Figure 7A and Dataset S7). In this dataset of 47 fibroblast populations, we observed three clear patterns of *HOX* gene expression among distal, trunk, and nondermal fibroblasts that parallel boundaries of murine *HOX* gene expression patterns during development. Specifically, the expression of *HOXB* genes *(HOXB2, HOXB4, HOXB5, HOXB6, HOXB7,* and *HOXB9)* was limited to the trunk and nondermal samples, whereas *HOXD4* and *HOXD8* were expressed exclusively in the trunk and proximal leg samples. This is reminiscent of the role of these genes in setting up the anterior-posterior axis, patterning of the body and lung development during embryogenesis in both the mouse and humans models [17,18]. *HOXA13,* a regulator of distal fates during embryonic development (reviewed in [19]), was expressed exclusively in adult fibroblasts isolated from distal sites (feet, fingers, foreskin; Figure 7A).

Variation in *HOX* gene expression recapitulated many of the organizational features produced by grouping fibroblasts based solely on the variation of genes relative to the three anatomic divisions. Using hierarchical clustering based on the expression of homeodomain genes, 43 of 47 fibroblast samples were grouped into clusters that contained predominantly samples from the same anatomic origin (upper limb, lower limb, head and trunk, foreskin, and nondermal tissues; Figure 7A). Removal of the canonical *HOX* genes from the set of genes used for hierarchical clustering abrogated this organization, leaving 35 of 47 clustered by anatomic structure (the expected default based on using all genes or random small gene sets). These results confirmed a systematic variation in the expression of *HOX* genes in cultured fibroblasts corresponding to their anatomic site of origin.

We corroborated the site-specific expression of *HOX* genes in adult fibroblasts by several means. The RNA expression of several *HOX* genes in cultured cells was confirmed by reverse transcription PCR (Figure 7B and unpublished data). In addition, immunoblotting confirmed that HoxB4 protein was highly expressed in fetal lung fibroblasts (nondermal) but not in foreskin fibroblasts (Figure 7C). HoxA13 protein was highly expressed in foot fibroblasts and human foreskin tissue (both sites with “distal” gene expression patterns and HOXA13 mRNA expression) but not in thigh fibroblasts (proximal) (Figure 7D). Finally, immunofluorescence of human foreskin confirmed that approximately one third of the dermal fibroblasts expressed nuclear HoxA13 protein in vivo (Figure 7E). Dermal HoxA13 immunoreactivity did not occur in circular patterns that might be indicative of endothelial cells. HoxA13 immunoreactivity in foreskin epidermis was likely a nonspecific artifact: The epidermal staining was diffuse rather than nuclear, and immunoblotting confirmed HoxA13 expression in cultured foreskin fibroblasts but not in foreskin keratinocytes (Figure S4). In each of the instances tested, the differential mRNA expression we observed in cultured fibroblasts was paralleled by differential protein expression. Thus, adult fibroblasts systematically retain features of the embryonic HOX code in vitro and in vivo; these findings suggest that fibroblasts encode positional identities retained from embryonic development. Whether their expression program was actually established by fibroblast progenitors during embryogenesis, however, remains to be answered.

## Discussion

In this study, we analyzed the spatial organization of the global gene expression programs of 47 human fibroblast populations representing 43 anatomic sites. Our findings demonstrate on a large scale that fibroblasts have consistent and distinct gene expression programs depending on their anatomical origin. More important, our analysis identified several striking binary gene expression signatures that demarcated positional boundaries related to three anatomic divisions: one signature demarcating proximal and distal compartments of limbs, a second signature demarcating fibroblasts from the anterior or posterior half of the body, and a third expression signature distinguishing the dermal fibroblasts from those of nondermal origin. When the three binary gene expression signatures were integrated and used to classify fibroblasts spanning the body, they were able to group fibroblasts from this and other datasets by their anatomic origin. The site-specific variation in gene expression of fibroblasts is therefore not stochastic or idiosyncratic. Rather, the significant features of the anatomic specialization of fibroblasts can be accounted for by systematic differences in gene expression with respect to three broad anatomic divisions: anterior-posterior, proximal-distal, and dermal-nondermal.

This surprisingly simple spatial organization of fibroblast differentiation, even measured on a genome-wide scale, is supportive of a model of patterning based on position on a coordinate system. The generality of the coordinate system is suggested by the shared “distal” gene expression signature between hands and feet, spatially distant tissues that are unlikely to have shared local regulatory interactions. The distal gene expression signature was also characteristic of foreskin fibroblasts. The male genitalia is developmentally a distal structure; its extension requires some of the same genes used in limb outgrowth, such as the 5′ *HOXA* and *HOXD* genes [20]. Similarly, gene expression signatures characteristic of anterior or posterior origin were shared along the trunk and the limbs (Figure 2). Finally, cross validation analyses on held out samples and an independent dataset confirmed the generality of the anatomic divisions as important features in fibroblast gene expression.

One important caveat of the positional identity model is that the three anatomic divisions we identified each consists of a binary pattern, which is a far sparser segmentation of the body map than the number of segments along each axes during embryonic development. We suggest three possible explanations for this result. First, because subtle, systematic gradations in expression would require larger number of samples to detect, our results do not rule out the possibility there may be finer segmentation or a gradient of differentiation along each anatomical axis, similar to that in early development. Second, this study only examined one “cell type” (fibroblasts) in vitro, and it is possible that the positional information related to additional anatomic divisions is encoded in other cell types or requires heterotypic cell-cell interactions for its manifestation. A third possibility is that while an elaborate coordinate system is employed during embryogenesis, only a sparse coordinate system is maintained in adulthood for tissue homeostasis. The anatomic divisions that remain in adulthood might have distinct mechanisms for their maintenance. Our identification of specific gene expression signatures that demarcate the adult anatomic divisions should facilitate future studies to distinguish among these possible mechanisms.

A model of fibroblast differentiation based on positional identity has several potentially important implications. First, the anatomic organization of fibroblast gene expression programs suggests that fibroblasts could be an important source of molecular landmarks for site-specific differentiation. Classic embryologic transplantation and tissue recombination experiments have shown that reciprocal epithelial-mesenchymal interactions are critical for patterning many organs, such as skin, lung, gastrointestinal, and genitourinary systems (reviewed in [21]). In many instances, mesenchymal cells are able to dictate the position-specific fates that develop in the overlying epithelia, but the particular mesenchymal cell type that conveys positional information in each case is not fully understood. Fibroblasts appear to retain an anatomically specific gene expression program that could provide a coordinate system of positional identity, which is stably maintained in the absence of heterotopic signaling when in ex vivo culture. Other cell types may also have distinct forms of “positional memory” that are reflected in their gene expression programs. For example, skeletal muscle cells exhibit graded expression of select genes related to their rostrocaudal position [22,23], but it is unknown whether they also show gene expression variation along proximal-distal or other developmental axes. Blood vessels also extend throughout the body, and the gene expression patterns of endothelial cells also demonstrate consistent variation in a organ-specific manner [24]. As fibroblasts activate gene expression programs characteristic of their position in the body, they could in principle construct an identifiable extracellular matrix and express structural and cell surface proteins that provide site-specific signaling for a given anatomic origin (Figure 5B).

Second, conveyance of positional identity is also essential during wound healing and regeneration. We speculate that in addition to the well-known roles of fibroblasts in the synthesis of extracellular matrix for the tensile strength of wounds, fibroblasts provide important positional cues for scaling, connecting, and specifying the regenerated tissues. In regenerative therapies based on stem cells, fibroblasts may play similar instructional roles by providing the appropriate stem cell niche for directed stem cell differentiation [25].

Third, the organization of differentiated fibroblasts based on superimposed positional gene expression patterns strongly implicates epigenetic mechanisms that stably specify fibroblast differentiation. Indeed, important features of the embryonic pattern of *HOX* gene expression appear to be retained in this diverse sampling of cultured adult fibroblasts. A prime example is the selective expression of *HOXA13* in fibroblasts from developmentally distal structures such as fingers, feet, and foreskin, but not in fibroblasts just a few inches more proximal in the arm, leg, and trunk, respectively. *HOXB* and *HOXD* genes are expressed exclusively in the trunk/proximal leg, and internal organs. Because the expression pattern of *HOX* genes in these fibroblasts is systematically related to their anatomic site of origin, *HOX* genes are strong candidates as regulators of differentiation of adult fibroblasts. During development, *HOX* genes are first induced and achieve their nested, segmental pattern of expression in the somites; the dorsal region of each somite later gives rise to the dermis in a regional-specific manner [26]. These lineage-tracing studies and our observation of HoxA13 protein expression in a subset adult dermal fibroblasts in vivo are thus fully consonant and support the hypothesis that elements of the HOX code act as regulators of fibroblast differentiation.

The stability of these transcriptional pattern in vitro after long-term passage suggest that differentiated fibroblasts have a robust mechanisms to stably maintain the epigenetic information important for anatomically specific differentiation. Bernstein et al. [27] recently demonstrated that the chromatin of *HOX* loci are differentially modified in adult fibroblasts in a site-specific manner. In the future, functional studies with lineage-specific genetic manipulation will be needed to identify the specific roles of *HOX* genes in fibroblast differentiation and epithelial development. Our study provides a large collection of fibroblasts expressing a variety of *HOX* genes in which to study chromatin regulation. The accessibility and versatility of fibroblast culture techniques will make fibroblasts an excellent system in which to study the genetic and epigenetic mechanisms of positional identity. Moreover, this system may provide important insights into *HOX* gene regulation and the identity of HOX-regulated gene products in human cells.

Finally, while in the present study we have characterized the diversity of fibroblast cultured from grossly different anatomic sites, fibroblasts at a much finer histological detail, e.g., from different layers of the dermis and associated with different appendages (such as hair follicles or sweat glands) also have different functional properties. How these additional levels of fibroblast specialization are specified and the roles they play in shaping and maintaining the stromal architecture are important questions for future investigation.

## Materials and Methods

### Sample collection and cell culture.

Thirty-five primary human fibroblast cultures were established from skin of six donors from discarded normal tissue during surgical repair or from autopsy using a skin punch. The anatomic sites were mapped using a preset diagram based on bony landmarks of the donors. Fibroblasts were isolated from keratinocytes and endothelial cells as described [28]. Seven primary fibroblast cultures from the internal organs were obtained from Clonetics (Rockland, Maryland, United States). The three foreskin and two fetal lung samples are as described [13]. Fibroblasts were propagated in vitro for five passages; at 80% confluence cells were synchronized by serum deprivation in media containing 0.1% fetal bovine serum for 48 h.

### Microarray procedures.

Total RNA was purified from each cell culture using TRIzol according to the manufacturer's instructions (Invitrogen, Carlsbad, California, United States). A universal standard reference of RNA from pooled cell lines was used as an internal standard for quantitative measurements (Stratagene, La Jolla, California, United States). Total RNA was amplified using Message Amp II (Ambion, Austin, Texas, United States) and labeled as described [29]. Human cDNA microarray construction and hybridization were as described [29]. Primary data is available at the Stanford Microarray Database (http://genome-www5.stanford.edu).

### Analysis of gene expression data.

Unsupervised hierarchical clustering and Pearson correlation calculations of gene expression profiles were performed using the Agilent GeneSpring Analysis Platform (Silicon Genetics, Palo Alto, California, United States). SAM [30] was used to identify genes that were differentially expressed among fibroblasts from different anatomic sites and from different compartments within each anatomical structure.

### Gene expression centroid analyses.

We sought to distinguish fibroblasts from arm versus those from leg (Figure 4C). To assess the repetitive nature of the anterior and posterior division in different anatomic structures of the body, we used a centroid analysis to test the similarity of gene expression variation between anterior- and posterior-derived cells from two different comparisons. We took gene expression data from cells derived from the head, trunk, and nondermal samples and averaged the gene expression patterns of cells derived from above or below the umbilicus to create the anterior and posterior gene expression centroids, respectively. A centroid is the average gene expression pattern of all samples of a class. The gene expression profile of each limb-derived fibroblast was then compared to the anterior and posterior centroid for similarity using noncentered Pearson correlation.

Training and cross-validation of gene expression signatures that predict rough anatomic origin (Figure S2) were performed by removing ten samples from the dataset, and the anterior-posterior and proximal-distal centroids were relearned without these samples by analysis of variance (*p* < 0.01) for proximal-distal and (*p* < 0.001) anterior-posterior, which reflect the sample sizes used (smaller *p*-value for larger sample sets). Gene expression profiles of the held-out test samples were compared to the relearned anterior-posterior and proximal-distal gene centroids by Pearson correlation to yield a site prediction for the held-out samples. Correlation values were plotted with respect to the anterior and distal centroids (created from the average expression profile of anterior and distal samples).

### Intersection of local and global models to identify 337 optimal positional identifier genes.

To identify a minimal set of positional identifiers that reflected both the local and global models, we defined a subset of genes that are differentially expressed in both the local and global models. We intersected the sum of nonredundant genes derived in each of the local analyses (i.e., proximal versus distal in upper and lower limbs, anterior versus posterior, and dermal versus nondermal) with the 1,237 differentially expressed genes identified from the five-class global model in SAM. The intersection of these two approaches identifies genes that are differentially expressed within each anatomical structure and across all samples, thus are not biased by either approach. A total of 337 genes overlapped between the local analyses and the global model (Figure 5A).

### Bootstrap analysis of anatomic organization by random gene sets.

To estimate the significance of grouping fibroblast samples by site, we used a boot-strap method to compare the observed result with the performance of random sets of genes.

We used a random number generator to identify random sets of 337 genes from the 7,580 well-measured and variably expressed genes within this dataset. Expression pattern of each random set of the 337 genes was used to group fibroblasts samples using hierarchically clustering, and then we counted the minimum number of branches that would have to be moved to place all the samples into clusters composed exclusively of cells from the same anatomic regions. The five anatomic regions were upper limb, lower limb, head and trunk, foreskin, and nondermal tissues. The score of correct grouping is 47 (the total number of samples) minus the number of branch moves. Since our scoring was done with discrete integers, we fit the resulting histogram to a discrete Gaussian instead of a continuous Gaussian. The median correct grouping by 100 random gene sets is 35 of 47. The correct grouping of 47 of 47 fibroblast samples by the 337 selected genes is highly significant (*p <* 0.00017).

### Test of the 337 genes in an independent dataset.

To determine if these 337 genes were also informative of anatomic origin of fibroblasts that were not included in this dataset, we extracted data for these 337 genes from the Stanford Microarray Database of previously published fibroblast samples [13] that are mutually exclusive from the 47 samples used in this study. These samples were hierarchically clustered based on the expression values of the 337 positional identifier genes in the independent dataset.

### Use of *HOX* gene expression to organize fibroblasts.

We identified a set of genes encoding homeodomain transcription factors that were reliably measured in our dataset, yielding 42 genes including 12 *HOX* genes. We tested the expression pattern of these genes relative to anatomic sites by performing two-way hierarchical clustering of all samples based on the expression pattern of these 42 homeodomain genes. The contribution of *HOX* genes to this organization was tested by removing the 12 *HOX* genes from the list of 42 genes and repeating the two-way hierarchical clustering.

### RT-PCR, immunoblotting, and immunofluorescence.

RT-PCR was performed by reverse transcribing 2 μg of total RNA from culture 1 or culture 6 using the retroscript kit (Ambion). All reactions had a parallel mock RT with all components except reverse-transcriptase. PCR was performed using Taq mastermix (Qiagen, Valencia, California, United States) with the following primers: *HOXA13* forward: cttctaccaccagggctacg, HoxA13 reverse: gcagagtggacttccagagg. S15 primers were included in retroscript kit (Ambion). *HOXA13* expression was further validated in six other fibroblast sites (foreskin, two foot samples, two thigh samples, and fetal lung) using Taqman quantitative one-step RT-PCR (Applied Biosystems, Foster City, California, United States). Assay on demand primers for *HOXA13* (Assay ID: Hs00426284) were normalized to GAPDH (Assay ID: Hs99999905_m1) levels and relative abundance was calculated using a delta-delta threshold analysis as described previously [31].

Antibodies and immunofluorescence to assess the lineage composition of primary fibroblast cultures were as described [13]. Cell lysates for immunoblotting were prepared from 5 × 10^5^ fibroblasts resuspended in 200 μl of lysis buffer (1% NP-40,15 mM Tris, and 150 mM NaCl), electrophoresed, and probed with antibodies to HoxA13 (Aviva Biosciences, San Diego, California, United States) or *HOXB4* (Developmental Studies Hybridoma Bank, University of Iowa, Iowa City, Iowa, United States). In vivo validation of HoxA13 expression was performed using 6-μm cryosections of human foreskin that were fixed with cold 100% acetone and blocked with 10% horse serum in PBS for 1 h followed by 1-h incubation of *HOXA13* antibody (Aviva) diluted 1:150 in 2% horse serum and PBS. Alexa Fluor (Invitrogen) conjugated secondary antibodies were incubated for 1 h on foreskin sections at 1:300 dilution in 2% horse serum and PBS.

## Supporting Information

Dataset S1 Data Used to Create Figure 1
(2.4 MB ZIP)

Dataset S2 Data Used to Create Figure 2
(1.2 MB ZIP)

Dataset S3 Data Used to Create Figure 3
(1.5 MB ZIP)

Dataset S4 Data Used to Create Figure 4
(497 KB ZIP)

Dataset S5 Data Used to Create Figure 5
(337 KB ZIP)

Dataset S6 Data Used to Create Figure 6
(299 KB ZIP)

Dataset S7 Data Used to Create Figure 7
(2 KB ZIP)

Figure S1 A Gene Expression Signature Distinguishes Fibroblasts from Dermal versus Nondermal Tissues The signature was identified by supervised analysis using the algorithm SAM.
(129 KB PDF)

Figure S2 Prediction of Anatomic Site of Origin by Gene Expression Signatures (A) Performance of anterior-posterior and proximal-distal gene centroids within the training set. Each fibroblast sample is positioned according to the correlation between its gene expression profile and the “anterior” and “distal” centorids (methods), respectively. Most fibroblasts from upper limb exhibit gene expression patterns with a positive correlation to the “anterior” centroid (left). Fibroblasts from finger and hands are distinguished by a positive correlation between their gene expression patterns and the distal centroid. Expression patterns of fibroblasts from the lower limb negatively correlate with the anterior centroid; distal and proximal lower limb samples are distinguished by a more positive or negative correlation to the distal centroid, respectively (middle). Most fibroblasts can be placed on the top or bottom half of the body based on gene expression by positively or negatively correlating with anterior centroid, respectively (right).
(B) Cross-validation of site prediction by gene expression signatures. We excluded ten samples (approximately 20%) from the dataset we used to train the anterior-posterior and proximal-distal gene expression centroids, and then used the gene centroid to predict the anatomic origin of these ten excluded fibroblasts samples. Overall, 80% of the predicted positional origins (anterior or posterior, proximal or distal) of the test fibroblast samples were correct.
(125 KB PDF)

Figure S3 Diversity of Site-Specific Fibroblast Gene Expression Is Not Significantly Captured by Exclusive Expression of Site-Specific Genes (A) Each of the 47 samples was assigned to an anatomic structure: arm (yellow), leg (red), trunk (green), foreskin (blue), and internal organs. We searched for genes that were exclusively expressed in each of the five structures.
(B) Heat map of 3,022 genes determined by SAM that are differentially expressed according to this model.
(C) Dendrogram of fibroblast samples based on similarity in expression of these 3,022 genes, as determined by hierarchical clustering. Samples are numbered and colored according to Figure 1. Thirty-five of the 47 samples were correctly grouped according to the anatomical structure of origin, a number no better than the performance of untrained or randomly selected groups of 337 genes. *Incorrectly grouped samples.
(350 KB PDF)

Figure S4 Immunoblot Analysis of Human Foreskin Keratinocytes and Fibroblasts for *HOXA13*
The HoxA13 antigen was not present in the epidermal keratinocytes but was present in cultured foreskin fibroblasts.
(100 KB PDF)

Table S1 Gene Ontology Categories that Were Enriched in the 337 Positional Identifiers Using GoMiner (http://discover.nci.nih.gov/gominer) the ontological categories represented in the list of 337 positional identifier genes were compared to the ontological categories represented by all 7,580 genes to find categories that were either overrepresented or underrepresented in the 337 positional identifier genes. All ontological categories that were either significantly enriched or unenriched (*p* < 0.02) are displayed with the their representative *p*-value.
(126 KB PDF)

## Abbreviations

FDR: false discovery rate
SAM: Significance Analysis of Microarrays
