# Supplementary figures and images for: Anatomic Demarcation by Positional Variation in Fibroblast Gene Expression Programs
Source: PLoS Genet. 2006 Jul 28;2(7):e119. doi: 10.1371/journal.pgen.0020119 (PMC1523235; doi:10.1371/journal.pgen.0020119)

Supplemental Figure 1

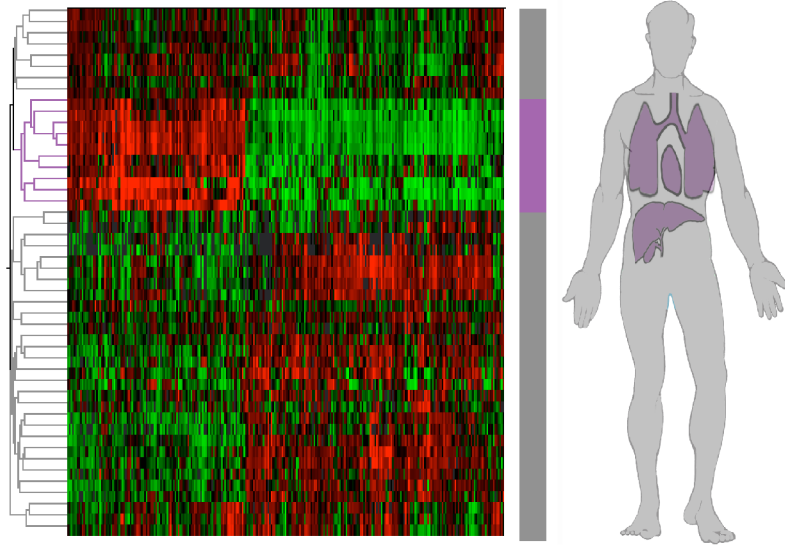

Supplement: Figure S1 — The signature was identified by supervised analysis using the algorithm SAM. (129 KB PDF) [file pgen.0020119.sg001.pdf]

Supplemental Figure 2

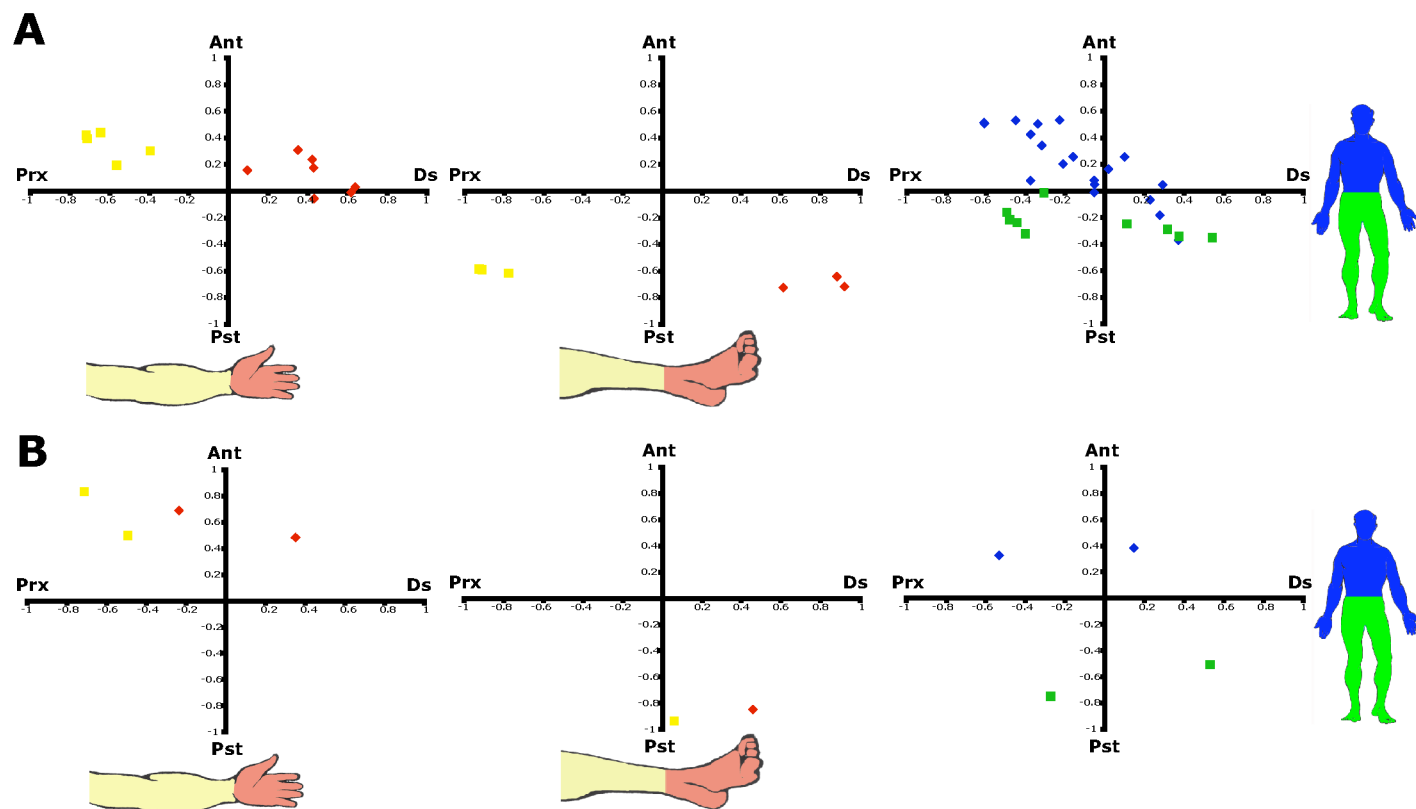

Supplement: Figure S2 — (A) Performance of anterior-posterior and proximal-distal gene centroids within the training set. Each fibroblast sample is positioned according to the correlation between its gene expression profile and the “anterior” and “distal” centorids (methods), respectively. Most fibroblasts from upper limb exhibit gene expression patterns with a positive correlation to the “anterior” centroid (left). Fibroblasts from finger and hands are distinguished by a positive correlation between their gene expression patterns and the distal centroid. Expression patterns of fibroblasts from the lower limb negatively correlate with the anterior centroid; distal and proximal lower limb samples are distinguished by a more positive or negative correlation to the distal centroid, respectively (middle). Most fibroblasts can be placed on the top or bottom half of the body based on gene expression by positively or negatively correlating with anterior centroid, respectively (right). (B) Cross-validation of site prediction by gene expression signatures. We excluded ten samples (approximately 20%) from the dataset we used to train the anterior-posterior and proximal-distal gene expression centroids, and then used the gene centroid to predict the anatomic origin of these ten excluded fibroblasts samples. Overall, 80% of the predicted positional origins (anterior or posterior, proximal or distal) of the test fibroblast samples were correct. (125 KB PDF) [file pgen.0020119.sg002.pdf]

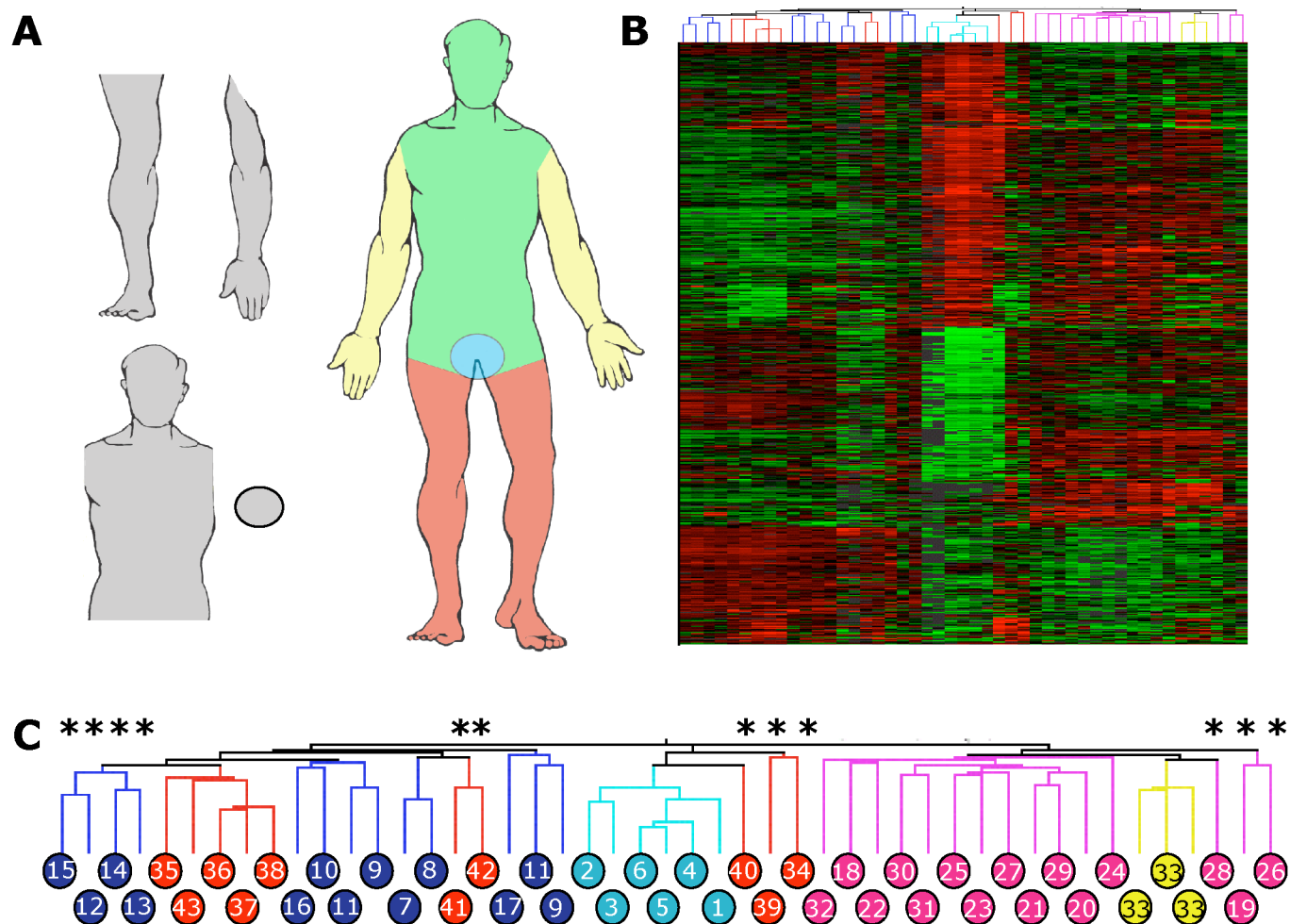

Supplement: Figure S3 — (A) Each of the 47 samples was assigned to an anatomic structure: arm (yellow), leg (red), trunk (green), foreskin (blue), and internal organs. We searched for genes that were exclusively expressed in each of the five structures. (B) Heat map of 3,022 genes determined by SAM that are differentially expressed according to this model. (C) Dendrogram of fibroblast samples based on similarity in expression of these 3,022 genes, as determined by hierarchical clustering. Samples are numbered and colored according to Figure 1. Thirty-five of the 47 samples were correctly grouped according to the anatomical structure of origin, a number no better than the performance of untrained or randomly selected groups of 337 genes. *Incorrectly grouped samples. (350 KB PDF) [file pgen.0020119.sg003.pdf]

Supplemental Figure 4

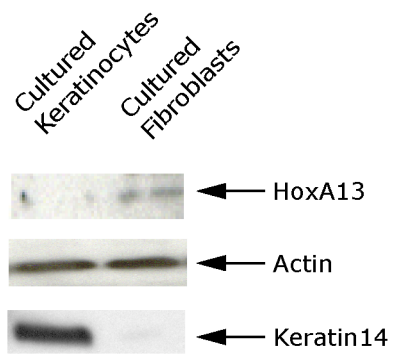

Supplement: Figure S4 — The HoxA13 antigen was not present in the epidermal keratinocytes but was present in cultured foreskin fibroblasts. (100 KB PDF) [file pgen.0020119.sg004.pdf]
