# Supplementary material for: Anatomic Demarcation by Positional Variation in Fibroblast Gene Expression Programs
Source: PLoS Genet. 2006 Jul 28;2(7):e119. doi: 10.1371/journal.pgen.0020119 (PMC1523235; doi:10.1371/journal.pgen.0020119)
Supplement: Table S1 — Using GoMiner (http://discover.nci.nih.gov/gominer) the ontological categories represented in the list of 337 positional identifier genes were compared to the ontological categories represented by all 7,580 genes to find categories that were either overrepresented or underrepresented in the 337 positional identifier genes. All ontological categories that were either significantly enriched or unenriched (p < 0.02) are displayed with the their representative p-value. (126 KB PDF) [file pgen.0020119.st001.pdf]

Supplemental Table 1

Category Name	P-Chn
plasma membrane	0.0038
regulation of transforming growth factor beta receptor signaling pathway	0.0041
transcription factor activity	0.0052
collagen	0.0057
extracellular matrix structural constituent	0.0066
protein binding	0.0081
G-protein coupled receptor protein signaling pathway	0.0088
extracellular matrix organization and biogenesis	0.0103
extracellular structure organization and biogenesis	0.0103
apicolateral plasma membrane	0.0114
ligand-dependent nuclear receptor activity	0.0118
nuclear membrane organization and biogenesis	0.0170
fever	0.0170
vacuolar membrane (sensu Magnoliophyta)	0.0170
oxidoreductase activity, acting on paired donors, with incorporation or reduction of ...	0.0170
dopamine beta-monooxygenase activity	0.0170
lactose synthase activity	0.0170
positive regulation of transforming growth factor beta receptor signaling pathway	0.0170
cathepsin E activity	0.0170
collagen fibril organization	0.0170
proteasome core complex, beta-subunit complex (sensu Eukaryota)	0.0170
mitotic nuclear envelope reassembly	0.0170
virus-host interaction	0.0170
viral envelope	0.0170
development of secondary sexual characteristics	0.0170
progesterone receptor signaling pathway	0.0170
vacuole (sensu Magnoliophyta)	0.0170
adenine nucleotide transporter activity	0.0170
7-alpha-hydroxysteroid dehydrogenase activity	0.0170
virion binding	0.0170
collagenase activity	0.0170
neutrophil collagenase activity	0.0170
purine nucleotide transporter activity	0.0170
